# Expansion of FasL-Expressing CD5^+^ B Cells in Type 1 Diabetes Patients

**DOI:** 10.3389/fimmu.2017.00402

**Published:** 2017-04-07

**Authors:** Ankit Saxena, Hideo Yagita, Thomas W. Donner, Abdel Rahim A. Hamad

**Affiliations:** ^1^Division of Immunology, Department of Pathology, Johns Hopkins University School of Medicine, Baltimore, MD, USA; ^2^Department of Immunology, Juntendo University School of Medicine, Tokyo, Japan; ^3^Department of Medicine, Johns Hopkins University School of Medicine, Baltimore, MD, USA

**Keywords:** type 1 diabetes, B cell, FasL (CD178), Fas (CD95), CD5, autoimmunity, gld, IL-10

## Abstract

Fas ligand drives insulitis in the non-obese diabetic mouse model of type 1 diabetes (T1D) and negatively regulates IL-10-producing (IL-10^pos^) CD5^+^ B cells in pancreata. Relevance of these phenomena to the human disease is poorly understood. Here, using splenocytes from T1D, autoantibody (Ab^+^), and non-diabetic (ND) human subjects, we show that a subpopulation of CD5^+^ B cells that is characterized by expression of FasL (FasL^hi^CD5^+^) was significantly elevated in T1D subjects, many of whom had significantly reduced frequency of IL-10^pos^CD5^+^ B cells compared to Ab^+^ subjects. The majority of FasL^hi^CD5^+^ B cells did not produce cytokines and were more highly resistant to activation-induced cell death than their IL-10^pos^CD5^+^ counterparts. These results associate expansion of FasL-expressing CD5^+^ B cells with T1D and lay the groundwork for future mechanistic studies to understand specific role in disease pathogenesis.

## Introduction

Type 1 diabetes (T1D) results from the destruction of insulin-producing pancreatic β cells by diabetogenic T cells. Such diabetogenic T cells are held in check by an array of tolerogenic mechanisms that maintain self-tolerance in the steady state (1). These mechanisms are mediated by a variety of immunoregulatory cell types and involve secretion of anti-inflammatory cytokines such as IL-10. In individuals at risk who progress to develop overt diabetes, regulatory mechanisms fail to curtail diabetogenic T cells that then attack and destroy β-cells, leading to hyperglycemia. This widely accepted model is built on extensive investigation of non-obese diabetic (NOD) mice and accumulated knowledge of the complex and often opposing roles of the immune cells in promoting and suppressing autoimmunity (2, 3). Whereas T cells are generally considered the main drivers of the diabetogenic process (4), B cells are equally important contributors. This is evident in the absence of significant insulitis and consequently overt hyperglycemia in B cell-deficient NOD mice (5–7). B cells are particularly important during the early inflammatory stage, including their role in enabling diabetogenic T cells to infiltrate pancreatic islets, resulting in the initiation and establishment of insulitis. This role is primarily attributed to the potent capacity of B cells to capture and present islet autoantigens to autoreactive T cells (8), but increasing evidence suggests a more complex relationship (9, 10). Given the pivotal role of B cells in the disease process, dissecting the various mechanisms used by B cells to affect autoimmunity is thus important for understanding disease pathogenesis and developing effective B cell-directed therapy.

The immunoregulatory mechanisms, like the pathogenic ones, are predominated by T cells and B cells. Among T cells, the Foxp3^+^ Treg cell, the best recognized immunoregulatory cell subtype, plays an essential role in maintaining insulitis in the benign state (11). Among B cells, there is no one definitive phenotype of the regulatory cells, but they are commonly enriched within the CD5^+^ subpopulation (12). Functionally, regulatory B cells are collectively identified by production of IL-10 (13). Immunoregulatory cells encounter and at least temporarily regulate autoreactive T cells at two main checkpoints in NOD mice. These checkpoints were elegantly illustrated in NOD mice bearing a monoclonal population of islet-reactive BDC2.5 TCR transgenic T cells (14). The first checkpoint involves events in pancreatic lymph nodes where autoreactive T cells are primed and acquire effector functions as well as the ability to infiltrate and reside within pancreatic islets. Failures at this checkpoint are marked by the development of benign insulitis that becomes destructive when immunoregulatory mechanisms at the second checkpoint fail, leading to the loss of β-cells and ensuing hyperglycemia. Identification of the molecules that trigger the failure of immunoregulation is very important for devising strategies to prevent diabetes and to halt disease progression, and is actively being pursued.

One such trigger in mice is the apoptosis-inducing FasL ligand (FasL), which is necessary for insulitis development. This is revealed by the arrest of the disease at a preinsulitis stage in NOD mice bearing homozygous gld mutations of FasL (NOD-gld/gld) or lpr mutations of its Fas receptor (NOD-lpr/lpr). This role of FasL is dominant as FasL-haploinsufficient NOD-gld/+ mice do not develop overt disease and only minor insulitis (15, 16). On the other hand, the primary physiological function of Fas/FasL interaction is induction of apoptosis of chronically activated lymphocytes and maintenance of immune homeostasis (17). Consequently, NOD-lpr/lpr and NOD-gld/gld develop massive, age-dependent T cell lymphoproliferation, yet remain insulitis- and hyperglycemia-free (18). To reconcile these apparently contradictory findings, it was suggested that Fas/FasL interactions were also essential for β cell apoptosis. Subsequent studies, however, found that the Fas/FasL system is not essential for autoimmune destruction of β cells (19, 20), and mechanisms by which the Fas death pathway regulates T cell islet infiltration remained obscured by the massive lymphoproliferation that develop in these mice. More recently, we and others have shown that the phenomena are dissociable as FasL haploinsufficiency (21) confers complete protection to NOD-gld/+ mice, which develop only minor insulitis but not lymphoproliferation (16, 22). Using these mice, we have shown that the gld mutation increases IL-10^pos^ CD5^+^ B cells in the pancreata and prevents insulitis by an IL-10-dependent mechanism (23). Very little, however, if anything, is known about the cell types that carry pathogenic FasL and their relationship to IL-10^pos^ CD5^+^ B cells. In addition, the relevance of these findings to human T1D had remained largely unexplored, despite the potential of FasL as a therapeutic target as it is not directly involved in T cell activation, and its modulation does not cause generalized immunosuppression (24).

We began addressing these questions in the present study. Our results associate expansion of FasL-expressing B cells with autoimmune diabetes and implicate them in the deletion of IL-10-producing Breg cells. The results suggest relevance of the long-known role of FasL in regulating susceptibility of NOD mice to autoimmune diabetes to the human disease and lay the groundwork for future mechanistic studies.

## Materials and Methods

### Human Donors and Tissue Procurement

Splenocytes used in this study were procured, recovered, and processed by the Network for Pancreatic Organ Donors with Diabetes (nPOD) and delivered in cryovials in liquid nitrogen and then kept at −80°C in our laboratory until used. Samples were from three cohorts: 14 T1D, 8 diabetes-free subjects with T1D-associated autoantibodies (Ab^+^), and 10 non-diabetic (ND) subjects. Clinical characteristics of donors are shown in Table 1.

**Table 1:** **Clinical and demographic details of donors provided by Network for Pancreatic Organ Donors with Diabetes**.

Id	Status	Autoantibody	Age	Duration	Gender	C peptide (ng/ml)	Hb1Ac	BMI
6180	T1D	GADA+ IA-2A+ ZnT8A+ mIAA+	27.1	11	M	<0.05	UK	25.9
6128	T1D	mIAA+	33.8	31.5	F	<0.05	UK	22.2
6138	T1D	mIAA+	49.2	41	F	<0.05	UK	33.7
6224	T1D	Neg	21	1.5	F	<0.05	UK	22.8
6152	T1D	ZnT8A(+)	29.6	12	F	<0.05	11.3	30.1
6204	T1D	GADA+ mIAA+	28	21	M	0.05	7.2	23.08
6211	T1D	GADA+ IA-2A+ ZnT8A+ mIAA+	24	4	F	0.05	10.5	24.4
6212	T1D	mIAA+	20	5	M	0.05	6.4	29.1
6236	T1D	GADA+ mIAA+	25	14	M	0.05	11.6	20.1
6237	T1D	GADA+ mIAA+	18	12	F	0.05	UK	26
6241	T1D	mIAA+	33	31	M	0.05	UK	18.4
6242	T1D	IA-2A+ mIAA+	39	19	M	0.05	UK	19.5
6244	T1D	mIAA+	34	28	M	0.05	5.9	23.8
6195	T1D	GADA+ IA-2A+ ZnT8A+ mIAA+	19.2	5	M	0.05		23.7
6170	Ab+	GADA+	34.4	NA	F	4.29	6.9	36.9
6123	Ab+	GADA+	23.2	NA	F	2.01	5.4	17.6
6158	Ab+	GADA+ mIAA+	40.3	NA	M	0.51	5.6	29.7
6184	Ab+	GADA+	47.5	NA	F	3.42	UK	27
6151	Ab+	GADA+	30	NA	M	5.49	UK	24.2
6156	Ab+	GADA+	40	NA	M	13.34	UK	19.9
6181	Ab+	GADA+	31.9	NA	M	0.61	UK	21.9
6171	Ab+	GADA+	4.3	NA	F	8.95	UK	14.8
6179	ND	Neg	21.8	NA	F	2.74	UK	20.7
6160	ND	Neg	22.1	NA	M	0.4	5.2	23.9
6131	ND	Neg	24.2	NA	M	1.01	UK	24.8
6140	ND	Neg	38	NA	M	11.1	6	21.7
6172	ND	Neg	19.2	NA	F	8.02	5.4	32.4
6165	ND	Neg	46	NA	F	4.45	UK	25
6229	ND	Neg	31	NA	F	6.23	5.5	26.9
6234	ND	Neg	20	NA	F	6.89	5.8	25.6
6174	ND	Neg	20.8	NA	M	3	UK	19.5
6178	ND	Neg	25	NA	F	4.55	UK	27.5

*T1D, type 1 diabetes; Ab^+^, autoantibody positive without diabetes; ND, non-diabetic and no autoantibody donors; UK, unknown; IA-2A, islet antigen-2 antibody; GADA, glutamic acid decarboxylase antibody; ZnT8A, zinc transporter 8 autoantibody; mIAA, microinsulin autoantibody*.

### Processing and Analysis Strategy of Human Samples

To minimize any effect of inter-experimental variation and maintain consistency of gating used to identify positive subpopulations in different diabetic, Ab^+^, and ND subjects, we performed our analysis by matching one T1D sample with at least one Ab^+^ and/or ND sample in each experiment, and the latter were used to draw gates to reduce any bias that could have resulted from analyzing T1D separately from Ab^+^ and ND samples. In total, we collected and performed statistical analysis in data pooled from 10 to 14 independent experiments. In each experiment, frozen splenocytes were thawed by using a standard operating procedure provided by nPOD. Briefly, cryovials were removed from liquid nitrogen, rapidly thawed with vigorous shaking for 2–3 min in a 37°C water bath, and added to pre-warmed medium (RPMI+ 10% FBS+ 50 U/mL Benzonase). Cells were centrifuged, washed, and counted by using trypan blue, and 1 × 10^6^ cells were used per application, unless stated otherwise. The absence of information regarding the absolute numbers of lymphocytes per spleen (we also received small pieces) made determination of absolute numbers of each subset per subject just a mere reflection of their frequency among acquired events, and for this reason we presented the data only as frequency of their indicated subsets.

### Staining Reagents

Fluorochrome-conjugated monoclonal antibodies (mAbs) specific for human antigens were obtained, unless stated otherwise, from BD Pharmingen (San Jose, CA, USA), BioLegend (San Diego, CA, USA), or eBioscience (San Diego, CA, USA). The following anti-human mAbs (clone) were used: FasL (NOK-1), Fas (DX2), CD19 (H1B19), CD5 (L17F12), CD40 (5C3), CD86 (IT2.2), CD20 (2H7), CD27 (LG.7F9), CD38 (HIT2), CD22 450 (S-HCL-1), CD10 (HI10a), CD1d (51.1), IL-10 (JES3-9D7), active caspase 3 (C92-605), IL-17 (eBio64DEC17), and IFN-γ (B27). Surface expression of human FasL was analyzed by using biotin-conjugated NOK-1 mAb. Previously described (25, 26) humanized anti-human FasL (RNOK203) was provided by Dr. Hideo Yagita of Juntendo University and Dr. Toshihiro Maeda of The Chemo-Sero Therapeutic Research Institute (Kaketsuken) in Japan.

### FACS Analysis of Surface Markers and Intracellular Cytokines

Single cell suspensions were stained with predetermined optimal concentrations of indicated mAbs. Samples were then washed and acquired using FACS LSRII flow cytometer and analyzed by Flow-JOV10 software. Three types of specificity controls were used. First, compensations were properly set using single-stained samples. Second, Fluorescence-Minus-One unstained samples and those stained with isotype controls were used to specifically gate positive cells and set up quadrants. Third, comparison of positive and negative cells in samples from donors (T1D, Ab^+^, and ND), and when appropriate, stimulated vs unstimulated samples provided internal controls. For intracellular staining, single cell suspensions were stimulated for 6 h with phorbol myristate acetate (PMA) (50 ng/mL) and ionomycin (500 ng/mL) in the presence of Golgi-Plug (2 mmol/L). Surface staining was followed by cell fixation, permeabilization, and intracellular staining with anti-IL-10, IL-17, TNFα, or IFN-γ mAb as indicated.

### Caspase 3 Apoptosis Assay

Splenocytes were stimulated with 10 µg/mL of affinity purified polyclonal goat F(ab’)2 anti-human IgM (Jackson ImmunoResearch) for 24 h at 37°C in the presence or absence of humanized anti-human FasL-neutralizing mAb (RNOK203) or the general caspase inhibitor Z-VAD-FMK (BD Biosciences). During the last 6 h of incubation, cultures were stimulated with PMA and ionomycin in the presence of Golgi-Plug, followed by surface staining and intracellular detection of IL-10 and active caspase 3 using specific mAbs. It is noteworthy that number of cells obtained per sample was limiting and did not allow a more direct and extensive analysis such as multi-color FACS analysis for FasL, Fas, CD5 on B cells without culture with anti-FasL mAb. Such analysis would have directly addressed whether IL-10-producing CD5^+^ B cells are specifically targeted for apoptosis.

### Statistical Analysis

Unless otherwise indicated, all data are shown as mean ± SEM. Statistical analysis was performed using the unpaired Mann–Whitney *U* test. A two-tailed value of *p* ≤ 0.05 was considered statistically significant. Statistical analysis and graph preparation were performed by using GraphPad Prism software (GraphPad Software, Inc., La Jolla, CA, USA).

## Results

### Detection of IL-10- and FasL-Expressing CD5^+^ B Subpopulations That Are Selectively Altered in Ab^+^ and T1D Subjects

**Figure 1:**
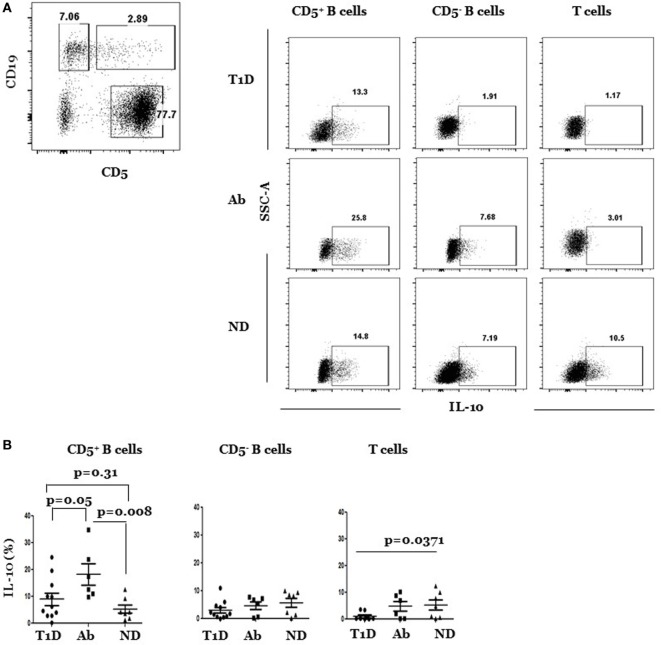
**IL-10^pos^ CD5^+^, but not IL-10^pos^ CD5^−^ B cells, are selectively enriched in Ab^+^ as compared to type 1 diabetes (T1D) and ND subjects**. Cryopreserved splenocytes from T1D, Ab^+^, and ND subjects were thawed and stimulated with phorbol myristate acetate and ionomycin, stained, and indicated subsets were gated as described under Section “Materials and Methods” and analyzed for IL-10 expression. **(A)** Left, dot plot shows gating strategy of CD5^+^ and CD5^−^ subsets of CD19^+^ B cells and CD5^+^ CD19^−^ T cells. Right, representative dot plots show intracellular expression of IL-10 by gated CD5^+^ and CD5^−^ B cells and T cells in different subjects. **(B)** Graphs show cumulative data pooled from 11 independent experiments. In each experiment, one T1D subject is paired with Ab^+^ and/or an ND subject. Each symbol represents a subject. Data were analyzed using the Mann–Whitney test and expressed as mean ± SEM; *p* < 0.05 is statistically significant.

It has long been known that FasL regulates insulitis and autoimmune diabetes development in NOD mice. In addition, our recent data showed that genetic or antibody inactivation of FasL leads to significant increases in IL-10-producing CD5^+^ B cells in the pancreata (23). To investigate relevance of these findings to the human disease, we analyzed IL-10- and FasL-expressing B and T cells in splenocytes from T1D, Ab^+^, and ND subjects. Overview of the gating strategy used to identify lymphocytes, exclude doublets and dead cells is outlined (Figure S1 in Supplementary Material). B cells are often analyzed as one population, yet they are heterogeneous and contain CD5^+^ B cells that are viewed as autoreactive and expanded in T1D patients [Figure S2 in Supplementary Material; Ref (27–30)]. CD5^+^ B cells also harbor the IL-10-producing regulatory subset. We therefore analyzed them separately from the CD5^−^ B cell subpopulation. CD5 is otherwise a pan marker of T cells. We stimulated samples with PMA/ionomycin and used the depicted gating strategy (Figure 1A) to distinguish between CD5^+^ CD19^+^ and CD5^−^ CD19^+^ B cells and CD5^+^ CD19^−^ T cells. Each subset was analyzed for IL-10 production in various subjects. Our results show that the frequency of IL-10^pos^ CD5^+^ B cells was significantly higher in Ab^+^ as compared to T1D subjects (Figures 1A,B). The difference was also significant when examined at the level of the absolute numbers of IL-10^pos^ CD5^+^ B cells (Figure S3 in Supplementary Material). On the other hand, the frequency, but not the absolute numbers, of IL-10^pos^ CD5^+^ B cells was higher in the Ab^+^ subjects than ND subjects (Figure S3 in Supplementary Material). There were no significant differences, however, in the frequency of IL-10-expressing CD5^−^ B cells (IL-10^pos^ CD5^−^ B cells) among the subjects in the three groups, excluding generalized upregulation of IL-10 in B cells of Ab^+^ subjects. Consequently and in line with the fact that CD5^−^ B cells comprised the bulk of B cells, the overall frequency of IL-10-expressing cells among total B cells (IL-10^pos^ B cells) was not statistically different among subjects in the three groups (Figure S4 in Supplementary Material). The frequency of IL-10^pos^ T cells, however, was significantly less in T1D as compared to ND, but not Ab^+^ subjects (Figure 1B). We concluded that there is selective expansion of IL-10^pos^ CD5^+^ B cells in Ab^+^ as compared to T1D subjects. The results extend the idea that the regulatory mechanisms in at-risk individuals, unlike in T1D progressors, are augmented and actively working to control islet autoreactivity (31, 32).

**Figure 2:**
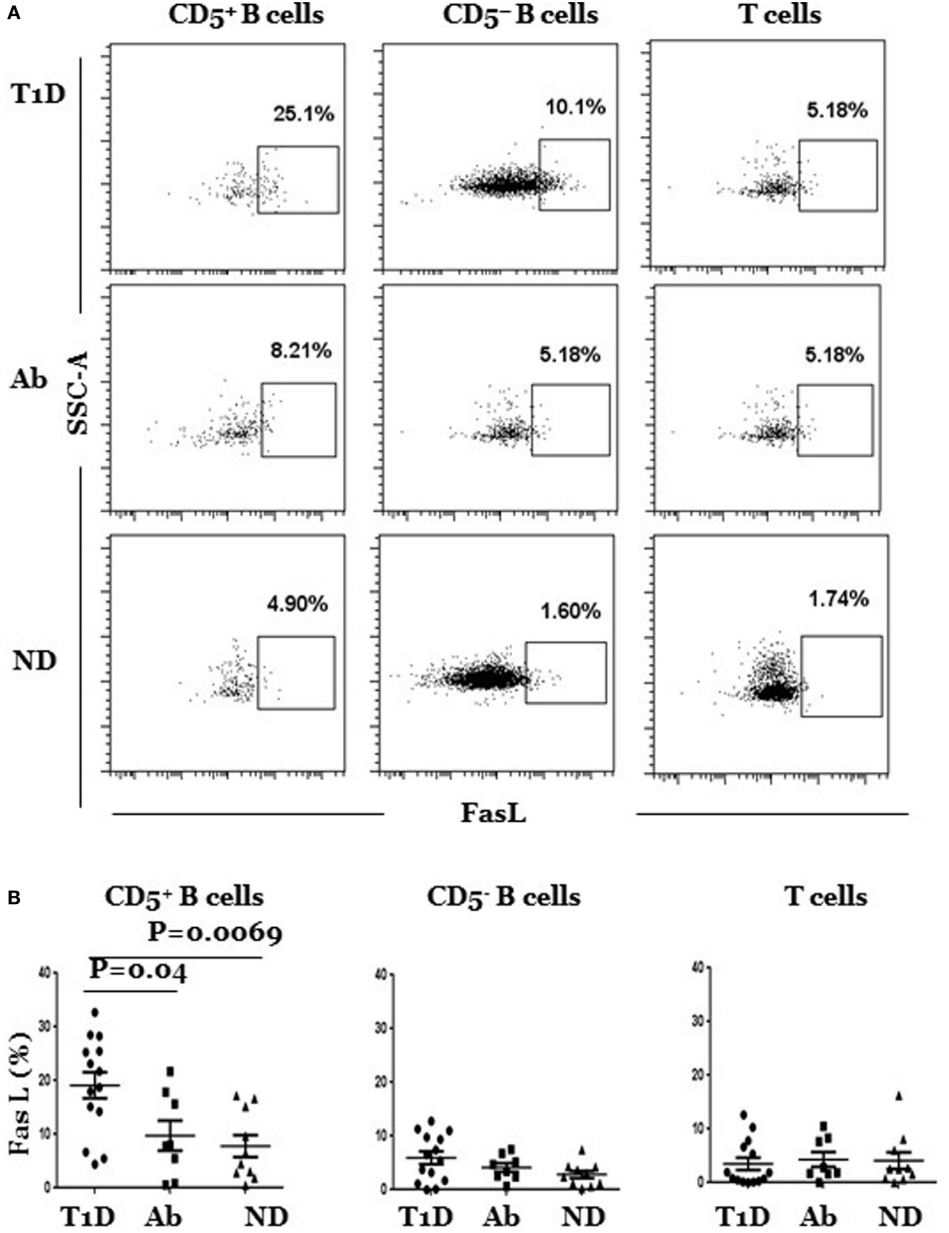
**FasL^hi^ CD5^+^, but not of FasL^hi^ CD5^–^ B cells, are selectively enriched in type 1 diabetes (T1D) as compared to Ab^+^ and ND subjects**. Cryopreserved splenocytes from T1D, Ab^+^, and ND subjects were thawed and surface-stained, and indicated subsets identified as described in Figure 1A were gated and analyzed for surface expression of FasL. **(A)** Representative dot plots show FasL expression by gated CD5^+^ and CD5^−^ B cells and T cells in different subjects. **(B)** Graphs show cumulative data pooled from at least 14 independent experiments. In each experiment, one T1D subject is paired with Ab^+^ and/or an ND subject. Each symbol represents one subject. Data were analyzed using the Mann–Whitney test and expressed as mean ± SEM; *p* < 0.05 is statistically significant.

Next, we determined whether FasL expression is dysregulated in B or T cells in any of the three subject groups. We detected significant differences that, as above, were limited to the CD5^+^ B cell subpopulation, albeit in the opposite direction, as the frequency of FasL-expressing CD5^+^ B cells (FasL^hi^ CD5^+^) was significantly higher in T1D as compared to Ab^+^ and ND subjects (Figure 2A). The frequency of FasL-expressing CD5^−^ B cells (FasL^hi^ CD5^−^) was generally low and slightly higher in T1D, but the difference among the groups did not reach statistical significance (Figure 2B). Likewise, the frequency of FasL-expressing T cells was comparable in the three groups, thereby excluding a generally dysregulated expression of FasL in T1D subjects (Figure 2B). In addition, CD5^+^ B cells in T1D, Ab^+^, and ND subjects had comparable surface expression of CD86 and CD40, confirming that the detected changes in IL-10 and FasL expression by these cells was specific and not due to generalized modulation of their surface profile (Figure S5 in Supplementary Material). Taken together, our results show IL-10^pos^ CD5^+^ B cells are selectively expanded in Ab^+^ subjects as opposed to selective expansion of FasL^hi^ CD5^+^ B cells T1D subjects.

### FasL^hi^ Cells Do Not Produce Cytokines and Have Reduced Susceptibility to Activation-Induced Cell Death (AICD)

**Figure 3:**
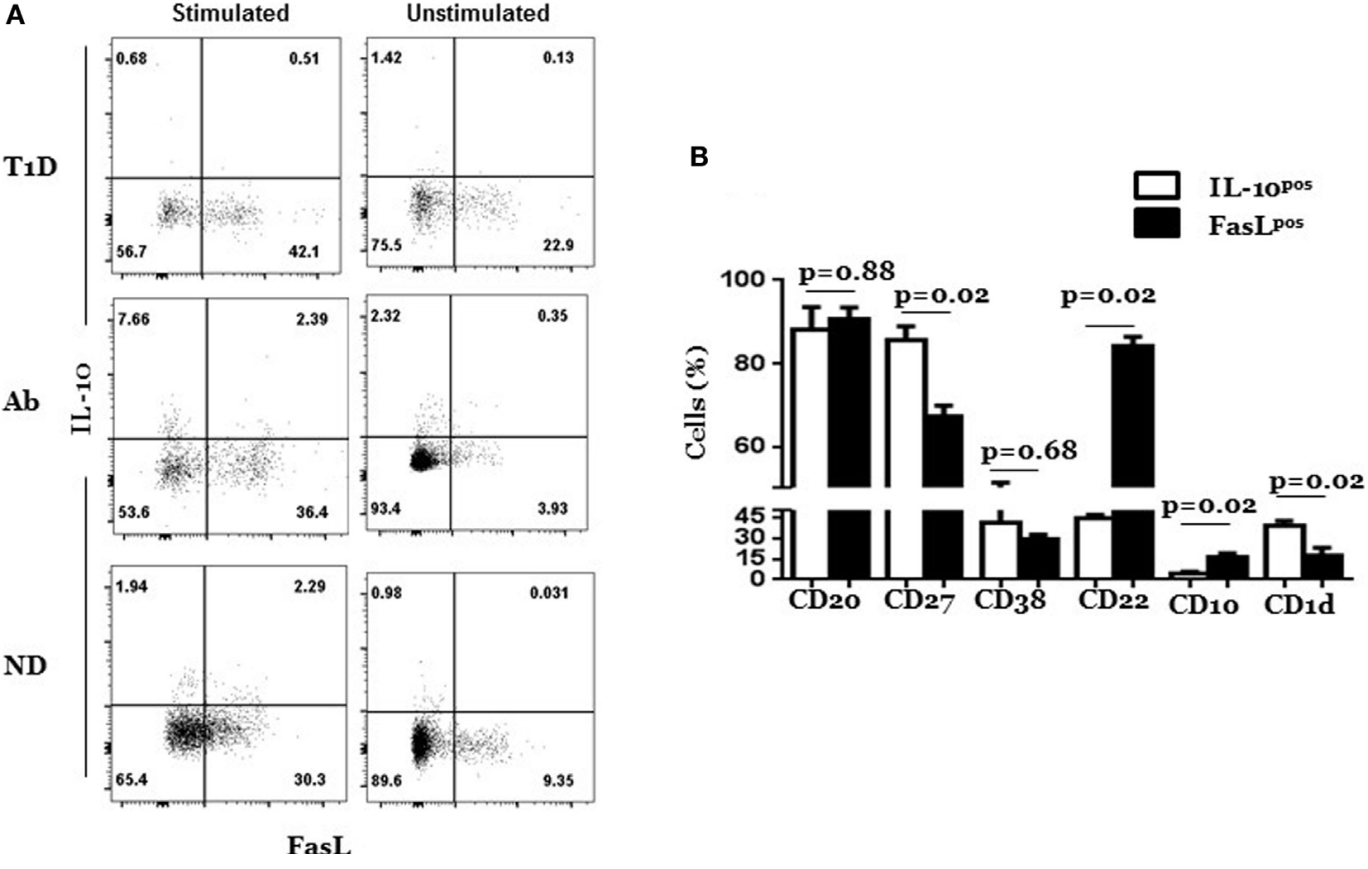
**Most FasL^hi^ CD5^+^ B cells do not produce IL-10 and exhibit less antigen-experience than IL-10^pos^ CD5^+^ B cells**. Cryopreserved splenocytes from type 1 diabetes (T1D), Ab^+^, and ND subjects were thawed and cultured in the presence or absence of phorbol myristate acetate and ionomycin for 6 h and analyzed simultaneously for surface expression of FasL and intracellular IL-10 as described under Section “Materials and Methods.” **(A)** Representative dot plots show expression of IL-10 and FasL by gated CD5^+^ B cells among stimulated and unstimulated cultures from T1D, Ab^+^, and ND subjects. Of note, the T1D dot plot was from the donor with the highest level of IL-10 expression so that the differential expression of FasL and IL-10 is better seen. **(B)** Graph shows expression of indicated surface molecules on gated IL-10^pos^ and FasL^hi^ subpopulations of CD5^+^ cells from stimulated cultures. Data are pooled from two independent experiments and four individually analyzed T1D subjects. Data were analyzed using the Mann–Whitney test and expressed as mean ± SEM; *p* < 0.05 is statistically significant.

The differential expansion of FasL^hi^ and IL-10^pos^ CD5^+^ cells in Ab^+^ and T1D indicated that they might represent two separate subpopulations. To directly test this notion, we simultaneously analyzed FasL and IL-10 expression at the single cell level in the CD5^+^ B cells from T1D, Ab^+^, and ND subjects by FACS after rapid PMA/ionomycin stimulation to upregulate IL-10. As predicted, we detected two distinct subpopulations that express either IL-10 or FasL in three types of donors (Figure 3A). The distinction, however, was not absolute as some cells were dual positive for FasL and IL-10. Nonetheless, the inability of FasL-expressing cells to produce cytokines was not limited to IL-10 or CD5^+^ B cells as it was the case with IFN-γ, TNFα, and IL-17 and for CD5^−^ B cells and T cells (Figure 3A; Figure S6 in Supplementary Material). Whether FasL^hi^ CD5^+^ B cells secrete cytokines other than those examined here cannot be formally ruled out and await to be determined. Differences between IL-10^pos^ and FasL^hi^ CD5^+^ cells extend to activation markers. IL-10^pos^ CD5^+^ B cells expressed significantly higher levels of CD1d and CD27 and lower levels of CD10 and CD22 than FasL^hi^ CD5^+^ B cells; but both expressed comparable levels of CD38 (Figure 3B; Figure S7 in Supplementary Material).

**Figure 4:**
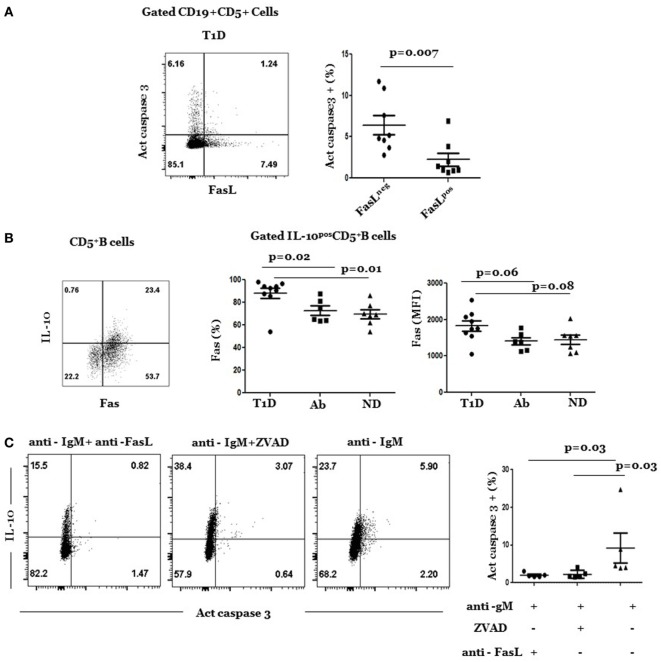
**Resistance of FasL^pos^ CD5^+^ B cells to activation-induced cell death**. **(A)** Cryopreserved splenocytes from type 1 diabetes (T1D) donors were thawed and stimulated with anti-IgM at 37°C for 24 h and analyzed for expression of active caspase 3 and FasL as detailed under Section “Materials and Methods.” Left, representative dot plot shows expression of active caspase 3 and FasL by gated CD5^+^ B cells. Right, graph shows cumulative data of frequency of caspase 3^+^ cells among gated FasL^pos^ and FasL^neg^ subpopulations of CD5^+^ B cells. Symbols represent individual donors. Data are expressed as mean ± SEM; *p* < 0.05 is statistically significant. **(B)** Percentage and levels (MFI) of Fas expression are significantly higher in IL-10^+^ CD5^+^ B cells in T1D than in Ab^+^ and ND subjects. Splenocytes from indicated donors were rapidly stimulated with phorbol myristate acetate/ionomycin and analyzed for intracellular IL-10 and Fas surface expression as described under Section “Materials and Methods.” Left, representative dot plot shows expression of IL-10 and Fas by gated CD5^+^ cells. Right, graphs show percentage (left) and mean fluorescent intensity (MFI) of Fas (right) on IL-10^+^ CD5^+^ B cells in subjects from the three groups. Data are expressed as mean ± SEM; *p* > 0.05 is statistically significant. **(C)** Cryopreserved splenocytes from T1D subjects were thawed and stimulated at 37°C with anti-IgM in the presence of FasL-neutralizing (RNOK203), the global apoptosis inhibitor, ZVAD, or in medium alone. After 24 h, cells in each culture were stained, and gated CD5^+^ B cells were analyzed for expression of active caspase 3 and IL-10. Left, dot plots show representative expression of active caspase 3 and IL-10 on gated CD5^+^ B cells under the different culture conditions. Right, graph shows cumulative data of percentage of IL-10^+^ CD5^+^ B cells that expressed active caspase 3 under each condition. Each symbol represents a subject (*n* = 5). Data was analyzed using Wilcoxon Matched pair sign rank test. Data are expressed as mean ± SEM; *p* < 0.05 is statistically significant.

FasL^hi^ CD5^+^ B cells expressed less Fas and were less susceptible to AICD than FasL^neg^ CD5^+^ B cells, including IL-10^pos^ CD5^+^ B cells. This was examined by the analysis of intracellular levels of active caspase 3 in anti-IgM-stimulated cultures. In this experiment, we stimulated splenic cultures with anti-IgM for 24 h and analyzed gated FasL^hi^ and FasL^neg^ subpopulations of CD5^+^ B cells for AICD. Most of apoptotic cells belonged to the FasL^neg^ subpopulation (Figure 4A) in which IL-10^pos^ cells resided. Therefore, in a second set of experiments, we examined whether AICD of IL-10^pos^ CD5^+^ cells was mediated by the Fas pathway. Consistent with this result, there was high Fas expression on IL-10^pos^ CD5^+^ B cells (Figure 4B, plot), which often denote sensitivity to AICD (33). The frequency of IL-10^pos^ CD5^+^ cells was significantly higher in T1D than in Ab^+^ and ND subjects (Figure 4B, graphs). On the other hand, mean level of Fas expression (MFI) was higher in T1D but was not statistically different than its levels in Ab^+^ and ND subjects. Moreover, apoptosis of IL-10^pos^ cells was inhibited by FasL-neutralizing mAb to an extent that was comparable to that induced by the pan caspase inhibitor, ZVAD (Figure 4C). It is noteworthy that cultures treated with anti-FasL mAb often reduced the overall frequency of IL-10-producing B cells, suggesting perhaps a role for FasL in regulating IL-10 production that is worthy of future investigation. Taken together, these results show that most FasL^hi^ CD5^+^ B cells did not produce any of examined cytokines and that they differed from IL-10^pos^ CD5^+^ B cells in their activation state and susceptibility to AICD.

In summary, our results extend the long-known role for FasL in regulating pathogenesis of autoimmune diabetes in NOD mice to the human disease by showing dysregulated homeostasis of FasL^hi^ CD5^+^ B cells in T1D subjects. Future studies using conditional knock mice that lack FasL only in B cells will determine whether FasL expression in B cells plays an essential role in driving the diabetic process.

## Discussion

The present study associates a subpopulation of B cells that is characterized by the expression of FasL and CD5 (FasL^hi^ CD5^+^ B cells) with autoimmune diabetes in humans. Furthermore, our finding that T1D subjects harbor significantly fewer IL-10^pos^ CD5^+^ B cells than Ab^+^ subjects (Figure 4) confirms and offers plausible explanation for the recent observations by Kleffel et al. (34) and Deng et al. (35) of decreased frequency of IL-10^pos^ Breg cells in T1D. We speculate that FasL^+^ CD5^+^ B cells could be involved in the deletion of IL-10^pos^ CD5^+^ B cells. This notion is consistent with our previous findings that genetic inactivation of FasL in NOD mice increases the frequency of pancreatic IL-10^pos^ B cells and prevents insulitis development by an IL-10-dependent mechanism (23). Furthermore, paucity of IL-10^pos^ CD5^+^ B cells in NOD-wt mice is rapidly reversed by antibody blockade of FasL (23). The results begin to show relevance of the well-known pathogenic roles of functional Fas/FasL interactions in driving autoimmune diabetes in the NOD mouse model to human T1D. The results lay the foundation for developing strategies that target FasL for therapeutic purpose.

Historically, investigation of Fas-mediated apoptosis has been focused on T cells (36). Thus, identifying B cells as a critical source of pathogenic FasL appeared surprising. However, Fas-mediated apoptosis is a well-established deletion mechanism of autoreactive B cells (37, 38). Breakdown in this tolerance mechanism causes generalized expansion of B cells and production of autoantibodies in mice bearing homozygous loss-of-function gld or lpr mutations (39). Moreover, conditional deletion of Fas in B cells allows autoreactive B cells to escape checkpoints and quality control mechanisms in germinal centers (37, 38). Expansion of IL-10^pos^ CD5^+^ B cells in NOD-gld/+ mice or after injection of NOD-wt mice with FasL-neutralizing mAb suggests a direct role of Fas-mediated apoptosis in regulation of IL-10^pos^ CD5^+^ B cell homeostasis (23). In relevance to the human disease, our present results show that IL-10^pos^ CD5^+^ B cells in T1D subjects expressed significantly higher levels of Fas than in Ab^+^ and ND subjects, hence enhanced susceptibility to apoptosis (Figure 4B). We also must acknowledge that our data are indirect, and hopefully future experiments will assess this possibly directly at the single cell level.

It is clear that both FasL^hi^ CD5^+^ B cells and IL-10^pos^ CD5^+^ B cells have tinges of autoreactivity that could be subjecting them to Fas-mediated regulation. A primary indicator is the expression of CD5, which is usually associated with high self-reactivity, not only in B cells but also in T cells (40). Furthermore, FasL^hi^ CD5^+^ B cells and IL-10^pos^ CD5^+^ B cells were the only B cell subpopulations that were modulated up and down in manners that correlated with islet autoimmunity in both humans and mice. In contrast, there were no appreciable changes in frequencies of FasL^hi^ or IL-10^pos^ CD5^−^ B cells (Figures 1 and 2). IL-10^pos^ CD5^+^ B cells also expressed high levels of CD27 and CD1d (Figure 3B). CD27 is a general marker of memory B cells (41), and CD27^+^ B cells are more susceptible to apoptosis than CD27^−^ B cells in common variable immunodeficiency (42) and lupus patients (43). In addition, high expression of CD1d fits well with our characterization of IL-10^pos^ CD5^+^ subpopulation as regulatory B cells (12). In contrast, FasL^hi^ CD5^+^ B cells expressed high level of CD22 (Figure 3B). CD22 is a negative regulator of BCR signaling that diminishes B cell activation, and CD22-sufficient B cells are shown to be more resistant to anti-IgM-induced apoptosis than CD22-deficient B cells (44). Thus, phenotypic profiles of IL-10^pos^ CD5^+^ B cells and FasL^hi^ CD5^+^ B cells support the premise of this study that IL-10^pos^ CD5^+^ B cells are more antigen-experienced and likely more prone to Fas-mediated apoptosis than their FasL^hi^ CD5^+^ B counterparts.

In summary, the elevated frequency of FasL^hi^ CD5^+^ B cells in T1D subjects extends our previous findings to the human disease and begins to shed new light into a previously unappreciated dysregulation of the Fas pathway in T1D subjects.

## Ethics Statement

Additional information about procurement procedures, surgical processing, and recovery protocols are detailed in a recent review by Pugliese et al. (45) and at http://www.jdrfnpod.org/. The study was approved by the Johns Hopkins Medicine Institutional Review Board (JHM IRB).

## Author Contributions

AS did the experiments and analyzed the data. AH developed the hypothesis, designed experiments, interpreted the data, and wrote the manuscript. TD contributed to the genesis of the hypothesis and data interpretation and revised the manuscript. HY provided reagents and critically revised the manuscript.

## Conflict of Interest Statement

The authors declare that the research was conducted in the absence of any commercial or financial relationships that could be construed as a potential conflict of interest.
