# Supplementary material for: Expansion of FasL-Expressing CD5+ B Cells in Type 1 Diabetes Patients
Source: Front Immunol. 2017 Apr 7;8:402. doi: 10.3389/fimmu.2017.00402 (PMC5383713; doi:10.3389/fimmu.2017.00402)
Supplement: Supplementary file 1 [file Data_Sheet_1.PDF]

Supporting Information

Ankit Saxena[1], Hideo Yagita[2], Thomas W Donner[3], and Abdel Rahim A. Hamad[1,3, #]

[1]Division of Immunology, Department of Pathology, Johns Hopkins University School of Medicine, Baltimore MD 21205

[1]Department of Immunology, [2]Juntendo University School of Medicine, Tokyo, Japan

[3]Department of Medicine, Johns Hopkins University School of Medicine, Baltimore MD 21287

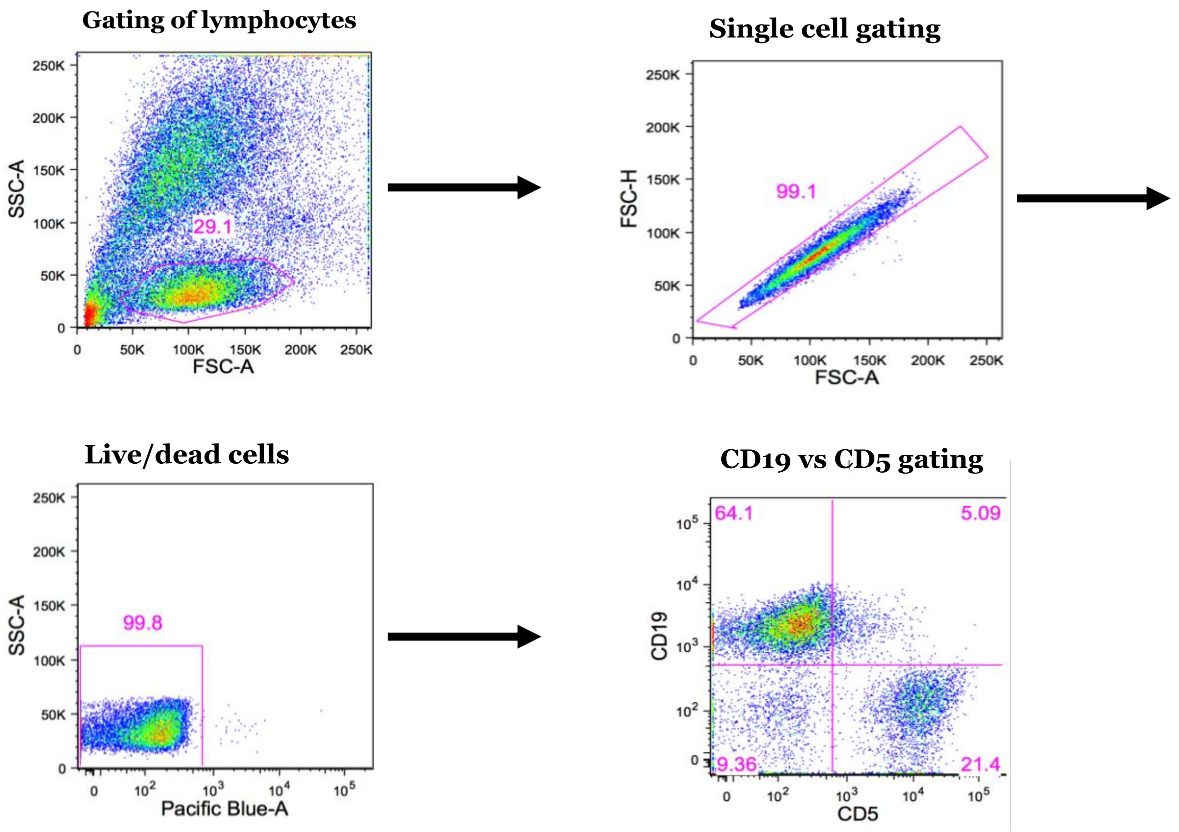

Supplementary Fig. 1. Outline of gating strategy. Single cell suspensions from cryopreserved splenocytes were stained for live dead marker and CD19, CD5 and relevant markers. **Dot plots** show (o'clock wise) gating of lymphocytes using FSC versus SSC, followed by single cell gating, and live/dead cells exclusion. Single live cells were then analyzed for CD19 and CD5.

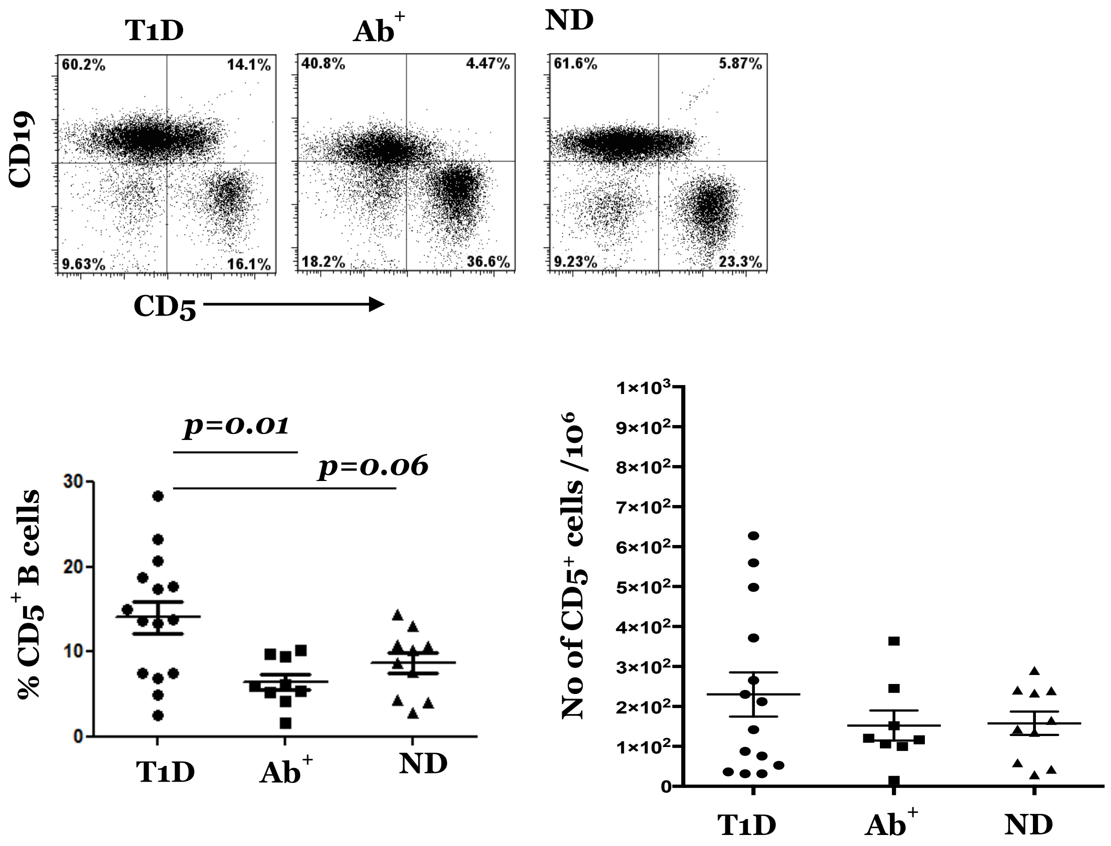

Supplementary Fig. 2. Frequency of CD5+ B cells of splenic B cells in different donors. Representative dot plots show expression of CD5 by splenic CD19+ B cells in T1D, Ab+, and ND subjects. **Left graph** shows cumulative data of percentages of CD5+ B cells in different subjects. **Right graph** shows absolute counts of CD5+ B cells per 10^6 cells. Each symbol represent one subject. Data analyzed using Mann-Whitney test. $p<0.05$ is statistically significant.

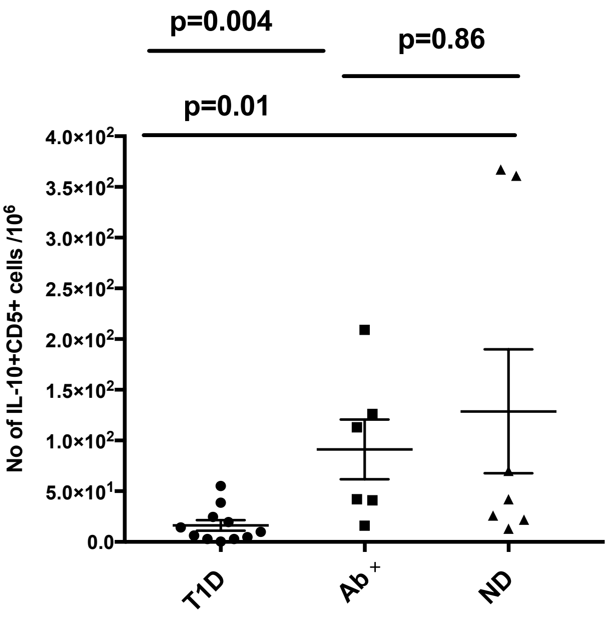

Supplementary Fig. 3. Absolute cell numbers of IL-10$^+$ CD5$^+$ B cells per 10^6 splenocytes are significantly higher in Ab$^+$ than T1D subjects. Absolute cell numbers were determined by multiplying the frequency of IL-10$^+$ CD5$^+$ B cells by total numbers of splenocytes determined using trypan blue exclusion. Statistical analysis was performed using Mann-Whitney test for unpaired samples and values $p<0.05$ considered significant.

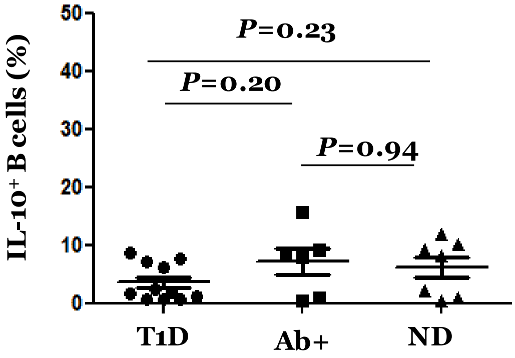

Supplementary Fig. 4. No evidence of generalized upregulation of IL-10 among B cells of Ab$^+$ subjects. Splenocytes from indicated subjects were stimulated with PMA and ionomycin and percentage of IL-10$^+$ cells among total CD19$^+$ B cells determined as described in Fig. 1. Each symbol represents one subject. Data analyzed using Mann-Whitney test. $p<0.05$ is statistically significant.

A

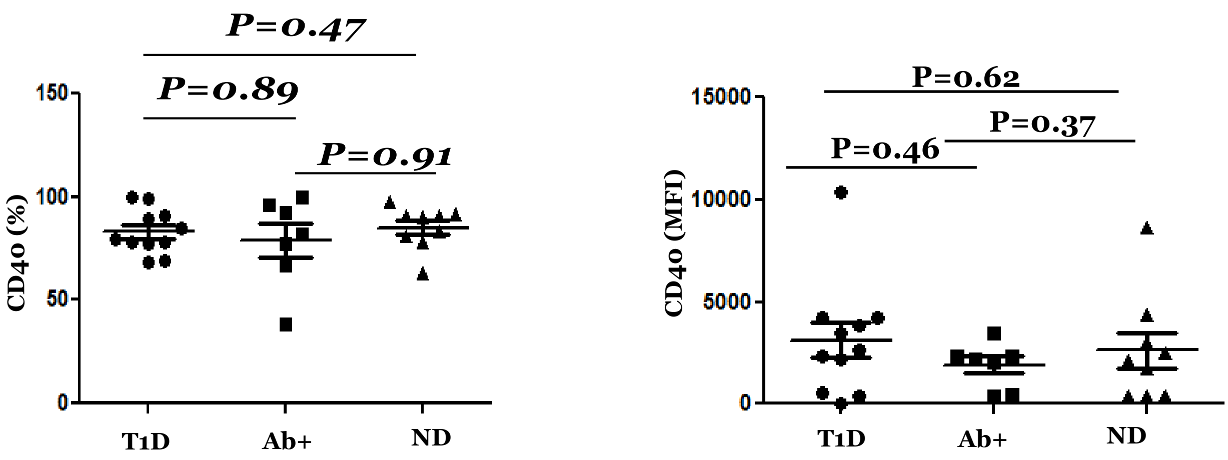

B

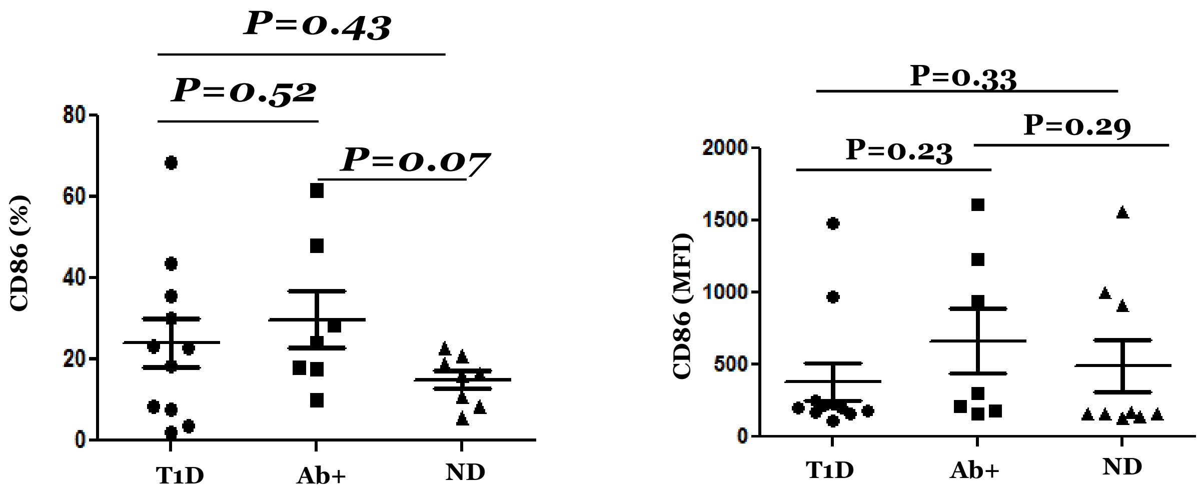

Supplementary Fig. 5. Comparable expression of CD86 and CD40 by CD5$^+$ B cells in T1D, Ab$^+$, and ND subjects. Cryopreserved splenocytes from indicated subjects were thawed, freshly stained and analysed for surface expression of CD86, CD40, CD19, and CD5 by FACS. Each dot represents a donor. **(A) Graphs** show percentages and MFI of CD40 by gated CD5$^+$ B cells. Graphs CD40. **(B) Graphs** show percentages and MFI of CD86 by gated CD5$^+$ B cells. Each dot represent one subject. Data are from T1D (n=11), Ab$^+$ (n=7), and ND subjects (n=8). Data analyzed using Mann-Whitney test. $p<0.05$ is statistically significant.

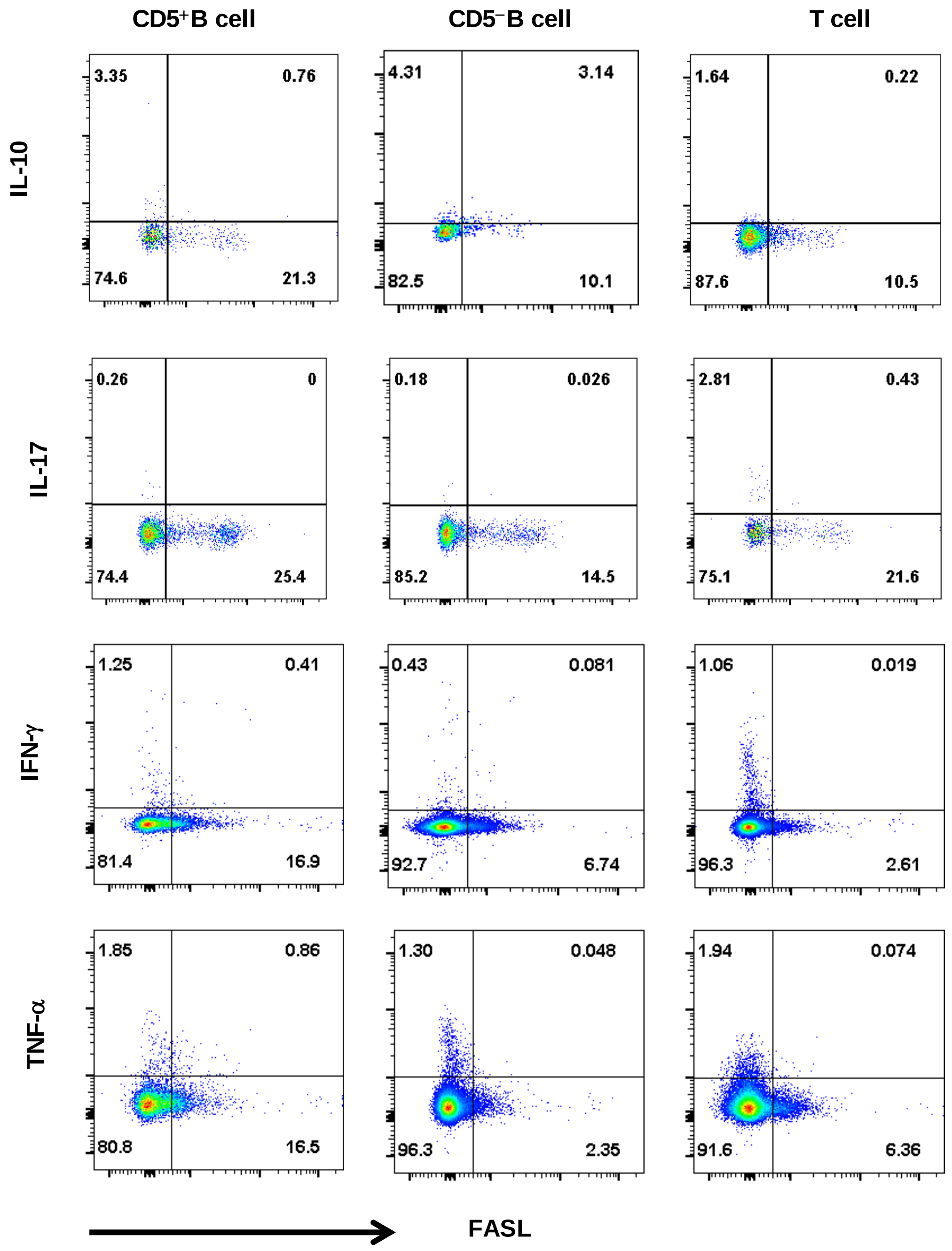

Supplementary Fig. 6. Most FasL-expressing cells do not produce indicated cytokines. Cryopreserved splenocytes from T1D subjects were stimulated with PMA and ionomycin and analyzed for surface FasL and intracellular IL-10, IFN-γ, IL-17 or TNFα, as in Fig. 3A. Representative dot plots show intracellular expression of each cytokine versus FasL in $CD5^+$ and $CD5^-$ B cells and T cells.

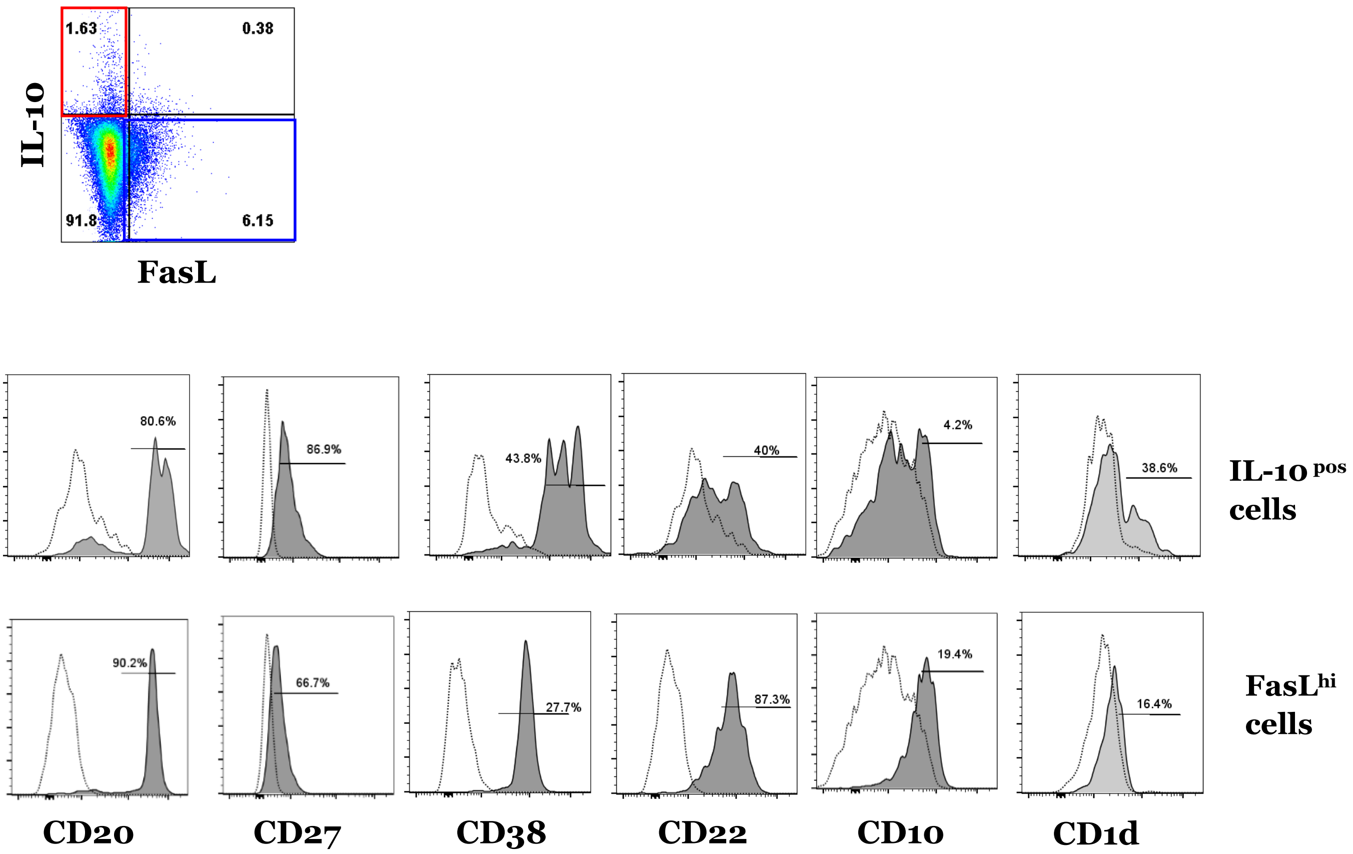

Supplementary Fig. 7. Representative staining of indicated surface markers by IL-10^{pos} (top panel) and FasL^{hi} (bottom panel) subpopulations of CD5^{+} B cells. Dot plot shows gating of IL-10^{pos} (outlined in red) and FasL^{hi} (outlined in blue) cells among CD5^{+} B cells. **Histograms** show representative expression of indicated surface molecules by gated IL-10^{pos} and FasL^{hi} subpopulations of CD5^{+} cells from samples described in Fig. 3B. Numbers indicate percentages of positive cells determined using FACS-minus-one staining for each subset to determine background levels. Analysis of cumulative data (Fig. 3B) shows that expressions of CD27, CD22, CD10, and CD1d were significantly different between the two subsets. On the other hand, no statistical differences in the expression of CD20 or CD38 was noted due to heterogeneity among analyzed subjects.

Table 1: Clinical and demographic details of donors provided by nPOD

Case Id	Donor Type	Autoantibody	Age	Diabetes duration	Gender	C peptide ng/ml	Hb1Ac	BMI
6180	T1D	GADA+ IA-2A+ ZnT8A+ mIAA+	27.1	11	M	<0.05	UK	25.9
6128	T1D	mIAA+	33.8	31.5	F	<0.05	UK	22.2
6138	T1D	mIAA+	49.2	41	F	<0.05	UK	33.7
6224	T1D	Neg	21	1.5	F	<0.05	UK	22.8
6152	T1D	ZnT8A(+)	29.6	12	F	<0.05	11.3	30.1
6204	T1D	GADA+ mIAA+	28	21	M	0.05	7.2	23.08
6211	T1D	GADA+ IA-2A+ ZnT8A+ mIAA+	24	4	F	0.05	10.5	24.4
6212	T1D	mIAA+	20	5	M	0.05	6.4	29.1
6236	T1D	GADA+ mIAA+	25	14	M	0.05	11.6	20.1
6237	T1D	GADA+ mIAA+	18	12	F	0.05	UK	26
6241	T1D	mIAA+	33	31	M	0.05	UK	18.4
6242	T1D	IA-2A+ mIAA+	39	19	M	0.05	UK	19.5
6244	T1D	mIAA+	34	28	M	0.05	5.9	23.8
6195	T1D	GADA+ IA-2A+ ZnT8A+ mIAA+	19.2	5	M	0.05		23.7
6170	Ab+	GADA+	34.4		F	4.29	6.9	36.9
6123	Ab+	GADA+	23.2		F	2.01	5.4	17.6
6158	Ab+	GADA+ mIAA+	40.3		M	0.51	5.6	29.7
6184	Ab+	GADA+	47.5		F	3.42	UK	27
6151	Ab+	GADA+	30		M	5.49	UK	24.2
6156	Ab+	GADA+	40		M	13.34	UK	19.9
6181	Ab+	GADA+	31.9		M	0.61	UK	21.9
6171	Ab+	GADA+	4.3		F	8.95	UK	14.8
6179	ND	Neg	21.8		F	2.74	UK	20.7
6160	ND	Neg	22.1		M	0.4	5.2	23.9
6131	ND	Neg	24.2		M	1.01	UK	24.8
6140	ND	Neg	38		M	11.1	6	21.7
6172	ND	Neg	19.2		F	8.02	5.4	32.4
6165	ND	Neg	46		F	4.45	UK	25
6229	ND	Neg	31		F	6.23	5.5	26.9
6234	ND	Neg	20		F	6.89	5.8	25.6
6174	ND	Neg	20.8		M	3	UK	19.5
6178	ND	Neg	25		F	4.55	UK	27.5

Abbreviations: T1D, type 1 diabetes; Ab^{+}, autoantibody positive without diabetes; ND, non-diabetic and no autoantibody donors. UK, unknown. IA-2A, islet antigen-2 antibody; GADA, glutamic acid decarboxylase antibody; ZnT8A, zinc transporter 8 autoantibody; mIAA, microinsulin autoantibody.
