# Supplementary material for: Expansion of FasL-Expressing CD5+ B Cells in Type 1 Diabetes Patients
Source: Front Immunol. 2017 Apr 7;8:402. doi: 10.3389/fimmu.2017.00402 (PMC5383713; doi:10.3389/fimmu.2017.00402)
Supplement: Supplementary file 2 [file Data_Sheet_2.PDF]

Supporting Materials

Ankit Saxena[1], Hideo Yagita[2], Thomas W Donner[3], and Abdel Rahim A. Hamad[1,3, #]

[1]Division of Immunology, Department of Pathology, Johns Hopkins University School of Medicine, Baltimore MD 21205

[1]Department of Immunology, [2]Juntendo University School of Medicine, Tokyo, Japan

[3]Department of Medicine, Johns Hopkins University School of Medicine, Baltimore MD 21287

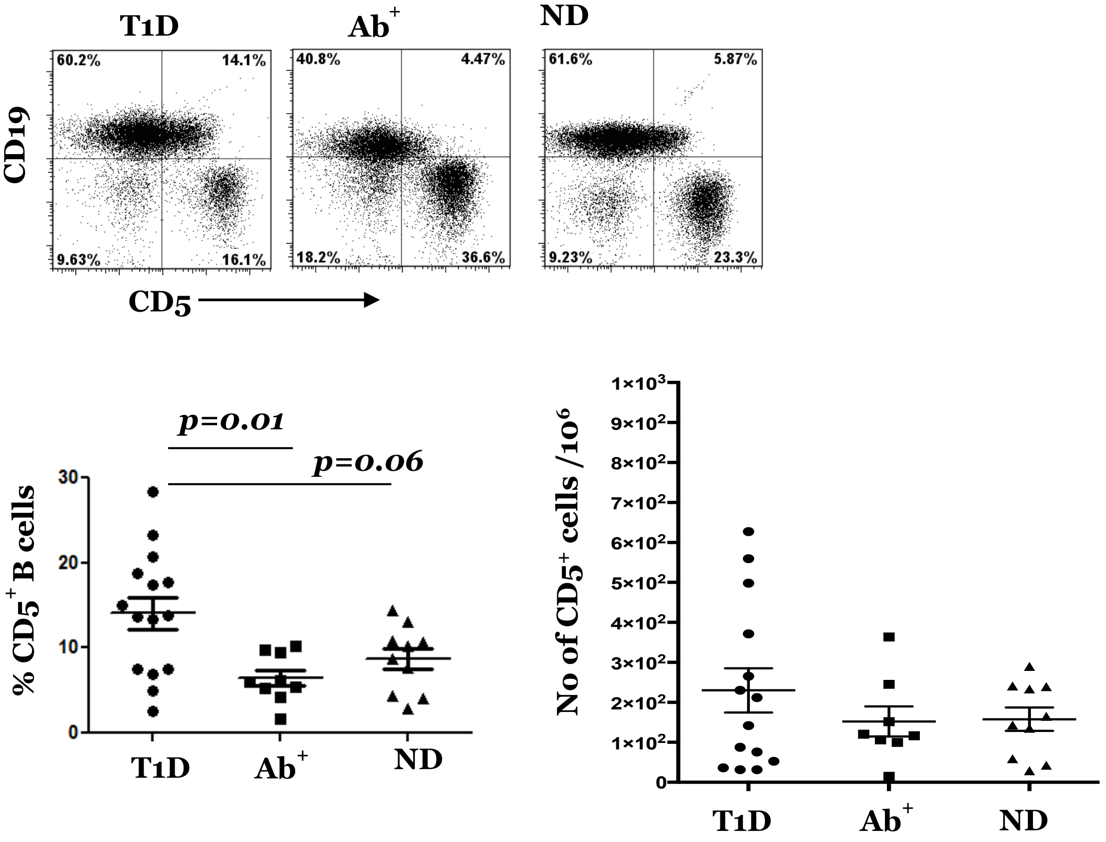

Supplementary Fig. 1. Frequency of CD5+ B cells of splenic B cells in different donors. Representative dot plots show expression of CD5 by CD19+ B cells in T1D, Ab+, and ND subjects. **Left graph** shows cumulative data of percentages of CD5+ B cells in different subjects. **Right graph** shows absolute counts of CD5+ B cells per 10^6 cells. Each symbol represent one subject. Data analyzed using Mann-Whitney test. $p<0.05$ is statistically significant.

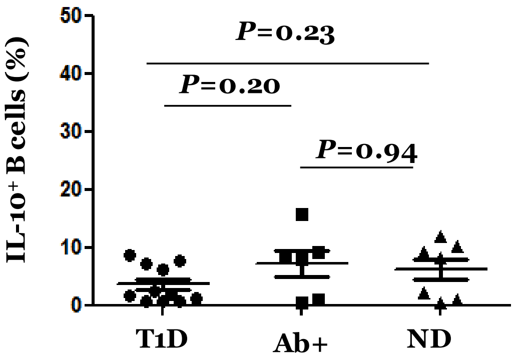

Supplementary Fig. 2. No evidence of generalized upregulation of IL-10 among B cells of Ab^{+} subjects. Splenocytes from indicated subjects were stimulated with PMA and ionomycin and percentage of IL-10^{+} cells among total CD19^{+} B cells determined as described in Fig. 1. Each symbol represents one subject. Data analyzed using Mann-Whitney test. $p<0.05$ is statistically significant.

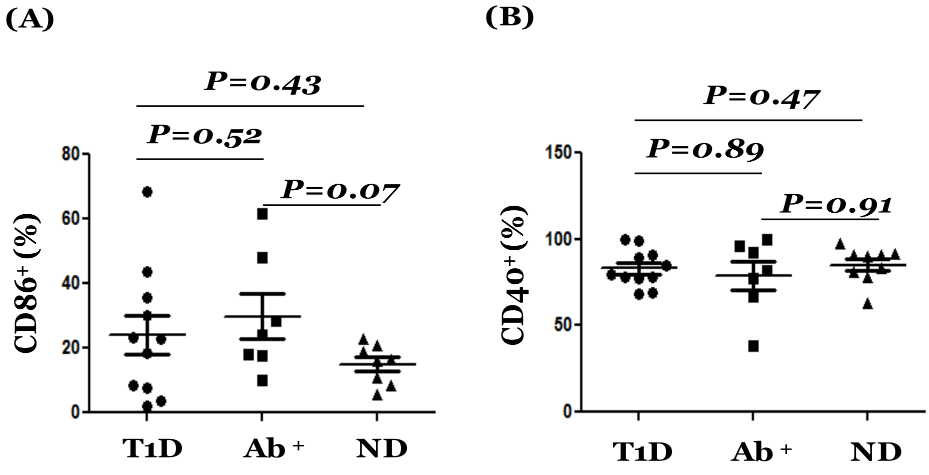

Supplementary Fig. 3. Comparable expression of CD86 and CD40 by CD5$^+$ B cells in T1D, Ab$^+$, and ND subjects. Cryopreserved splenocytes from indicated subjects were thawed, freshly stained and analysed for surface expression of CD86, CD40, CD19, and CD5 by FACS. Each dot represents a donor. Graphs show percentages of CD5$^+$ B cells that express CD86 **(A)** or CD40 **(B)** among splenocytes of T1D (n=11), Ab$^+$ (n=7), and ND subjects (n=8). Data analyzed using Mann-Whitney test. $p<0.05$ is statistically significant.

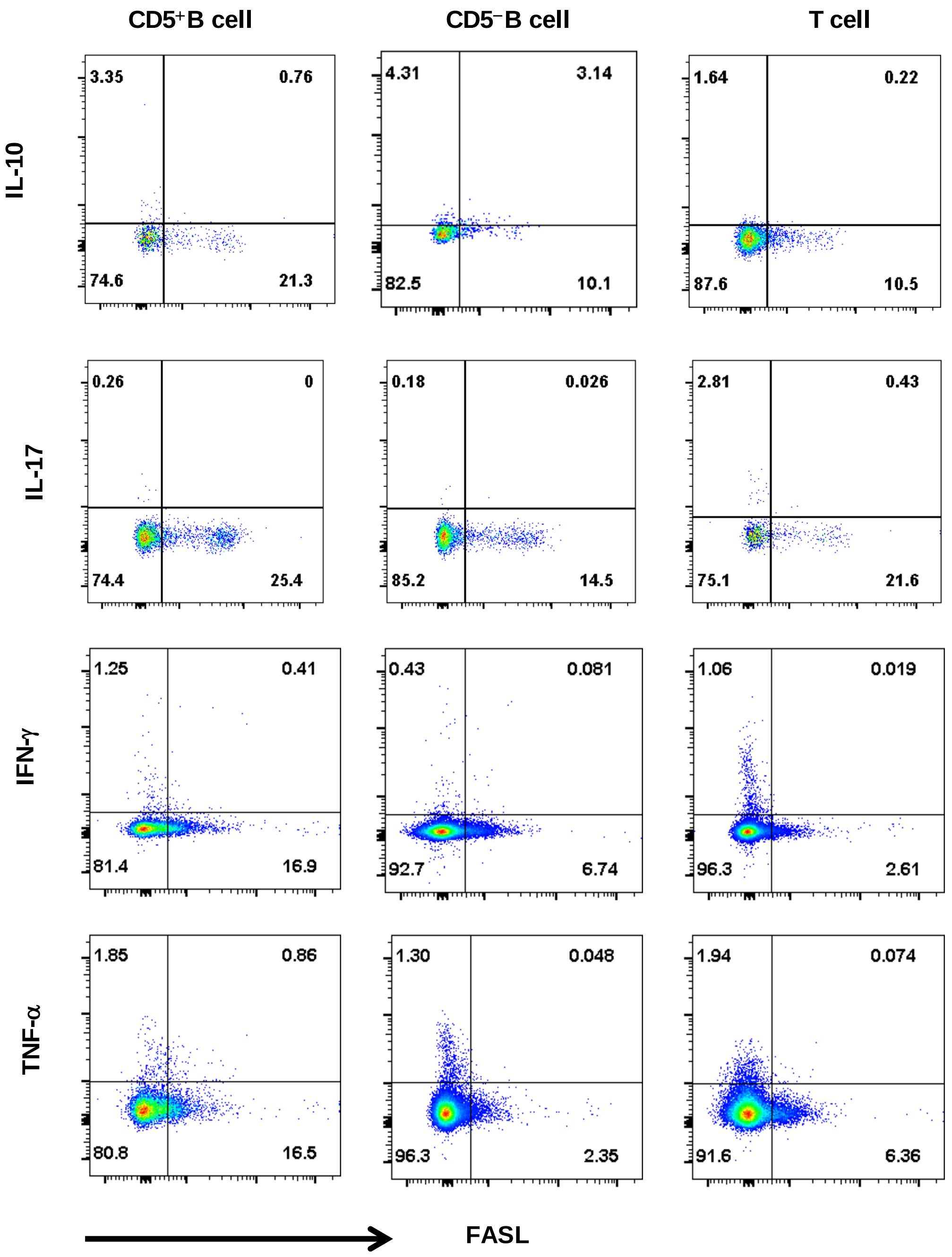

Supplementary Fig. 4. Most FasL-expressing cells do not produce indicated cytokines. Cryopreserved splenocytes from T1D subjects were stimulated with PMA and ionomycin and analyzed for surface FasL and intracellular IL-10, IFN-γ, IL-17 or TNFα, as in Fig. 3A. Representative dot plots show intracellular expression of each cytokine versus FasL in $CD5^+$ and $CD5^-$ B cells and T cells.

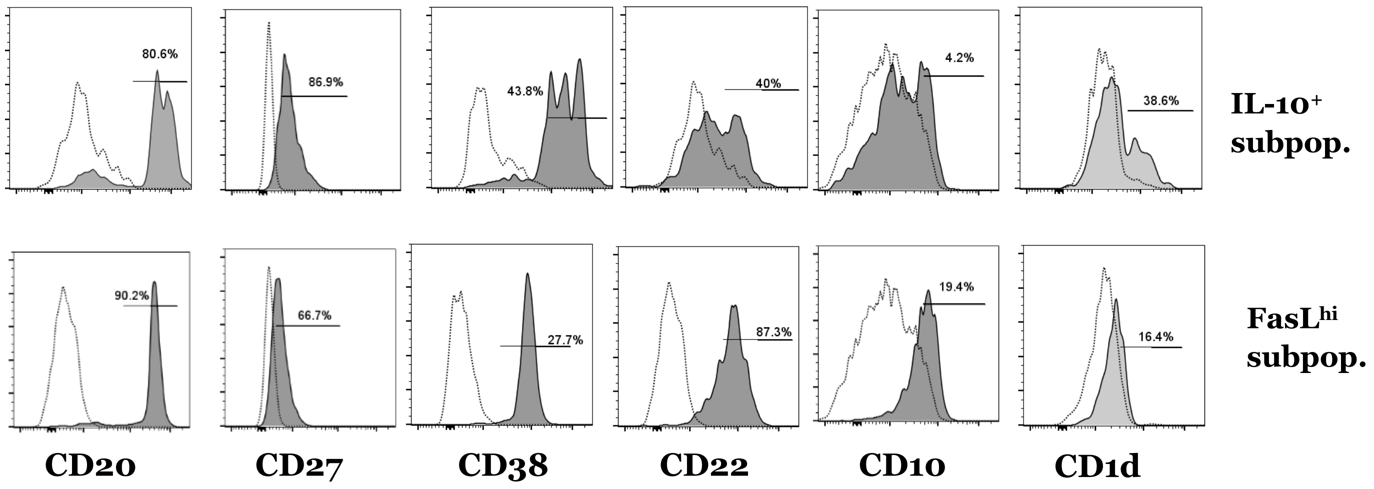

Supplementary Fig. 5. Representative staining of indicated surface markers by IL-10^{pos} (top panel) and FasL^{hi} (bottom panel) subpopulations of CD5^{+} B cells. Histograms show expression of indicated surface molecules on gated IL-10^{pos} and FasL^{hi} subpopulations of CD5^{+} cells from samples described in Fig. 3B. Numbers indicate percentage of positive cells, which were determined using FACS-minus-one staining for each subset to determine background levels. Analysis of cumulative data, Fig. 3B, shows that expression of CD27, CD22, CD10, and CD1d was significantly different between the two subsets. On the other hand, no statistical differences in expression of CD20 or CD38 due to heterogeneity among analyzed subjects.
